# Effect of Oxygen Vacancy on the Conduction Modulation Linearity and Classification Accuracy of Pr_0.7_Ca_0.3_MnO_3_ Memristor

**DOI:** 10.3390/nano11102684

**Published:** 2021-10-12

**Authors:** Yeon Pyo, Jong-Un Woo, Hyun-Gyu Hwang, Sahn Nahm, Jichai Jeong

**Affiliations:** 1 Department of Brain and Cognitive Engineering, Korea University, 145 Anam-ro, Seongbuk-gu, Seoul 02841, Korea; pyoyeon@korea.ac.kr; 2 KU-KIST Graduate School of Converging Science and Technology, Korea University, 145 Anam-ro, Seongbuk-gu, Seoul 02841, Korea; jong41187@korea.ac.kr (J.-U.W.); hghwang263@korea.ac.kr (H.-G.H.); 3 Department of Materials Science and Engineering, Korea University, 145 Anam-ro, Seongbuk-gu, Seoul 02841, Korea

**Keywords:** Pr_0.7_Ca_0.3_MnO_3_, memristor, resistive switching memory, convolutional neural network

## Abstract

An amorphous Pr_0.7_Ca_0.3_MnO_3_ (PCMO) film was grown on a TiN/SiO_2_/Si (TiN–Si) substrate at 300 °C and at an oxygen pressure (OP) of 100 mTorr. This PCMO memristor showed typical bipolar switching characteristics, which were attributed to the generation and disruption of oxygen vacancy (OV) filaments. Fabrication of the PCMO memristor at a high OP resulted in nonlinear conduction modulation with the application of equivalent pulses. However, the memristor fabricated at a low OP of 100 mTorr exhibited linear conduction modulation. The linearity of this memristor improved because the growth and disruption of the OV filaments were mostly determined by the redox reaction of OV owing to the presence of numerous OVs in this PCMO film. Furthermore, simulation using a convolutional neural network revealed that this PCMO memristor has enhanced classification performance owing to its linear conduction modulation. This memristor also exhibited several biological synaptic characteristics, indicating that an amorphous PCMO thin film fabricated at a low OP would be a suitable candidate for artificial synapses.

## 1. Introduction

Artificial neural networks inspired by biological neural networks have attracted much attention because they can perform complex computational tasks including learning and perception [1,2]. Conventionally, the von Neumann computing system has been used for artificial neural networks; however, this system has high operational power requirements [3]. Attempts to overcome this limitation led to the more recent proposal of the neuromorphic computing system, which is fault-tolerant and offers low-energy operation [1,4,5,6]. The fundamental concept of the neuromorphic computing system emerged from the biological brain, which performs the functions of perception, learning, and memory simultaneously [1]. The biological brain consists of neurons linked by synapses. Furthermore, a biological synapse is the cardinal component that performs learning and memory by controlling the synaptic plasticity [7,8,9,10,11,12,13]. Therefore, the fabrication of an artificial synapse with the ability to emulate biological synaptic characteristics is important to realize a neuromorphic computing system. Recently, various resistive switching random access memory (RRAM) devices have been considered as potential artificial synapses because of their simple structure, low operating power, and good interchangeability with the CMOS process [14,15,16,17].

RRAM memristors intended for application as artificial synapses should emulate important biological synaptic characteristics such as nonlinear transmission properties, transition from short-term plasticity to long-term plasticity (STP–LTP transformation), spike rate dependent plasticity (SRDP), and spike time dependent plasticity (STDP). In particular, a RRAM memristor would need linear conduction modulation to be suitable for application in a neuromorphic computing system to attain the learning accuracy and appropriate incremental switching [18,19,20,21,22,23]. If the conduction modulation of the memristor is not linear, an additional neuron circuit would be required to optimize the amplitude and duration of the input pulse [24,25]. Therefore, conduction modulation linearity is a highly important property that a RRAM memristor must possess for use in a neuromorphic computing system. The conduction modulation linearity of a RRAM memristor whose switching characteristics are determined by oxygen vacancy (OV) filaments is influenced by the enlargement (or disruption) of these OV filaments [18,26,27,28,29,30]. Changes in the size of the OV filaments in a RRAM memristor are generally explained by two reactions: a redox reaction (fast reaction), and OV diffusion (slow reaction) [18,26,29,30]. In general, a RRAM memristor has nonlinear conduction modulation because the enlargement (or disruption) of an OV filament is influenced by these two processes with their different reaction rates [18,26,29,30]. A RRAM memristor, in which the enlargement of the OV filaments is determined by only one of these processes, exhibits linear conduction modulation [18,26,29,30]. In the case of a small number of OVs, enlargement of the OV filaments is mainly determined by the diffusion of OVs, with linear conduction modulation as the result [18]. According to a previous study, the insertion of an additional SiO_2_ diffusion-limiting layer reduced the number of OVs in TaO_1−x_ film, resulting in linear conduction modulation in the TaO_1−x_ memristor [18]. In contrast, as the RRAM memristor contains numerous OVs, the redox reaction becomes a major growth mechanism of the OV filaments, leading to linear conduction modulation [29,30]. A CuO-added KNbO_3_ memristor and a NaNbO_3_ memristor annealed at reduced atmosphere showed linear conduction modulation because of the existence of a large number of OVs in the memristors [29,30]. The effects of OVs and thermal conductivity of the film on the linearity of the conduction modulation of ZnO-based memristors have also been studied [31].

Therefore, controlling the number of OVs in the memristor is considered to be highly important to ensure linear conduction modulation. In addition, the compliance current and the reset voltage were used to control the amount of OVs in the TiO_x_/Al_2_O_3_ devices [32], and the pre-spike was used to produce the OVs in the TiN/HfO_x_/Pd memristor [33].

Previously, Pr_0.7_Ca_0.3_MnO_3_ (PCMO) films have been deposited on various electrodes, and their RRAM properties have been extensively investigated [34]. Enhancing the RRAM properties and conduction modulation linearity of the PCMO RRAM devices was previously investigated using various top electrodes and device structures [35,36,37,38]. The switching properties of PCMO memristors were attributed to the interface controlled conduction mechanism [39,40]. In addition, growth and dissociation of the conducting filament was also suggested as a switching mechanism of PCMO memristors [41]. Moreover, both interface-type and filament-type switching mechanisms were simultaneously used to describe the switching characteristics of the PCMO memristor [42]. Hence, the switching properties of a PCMO memristor are considered to be considerably influenced by the deposition conditions of the film. Because a PCMO memristor generally exhibits good bipolar switching properties, the device is considered to be potentially capable of mimicking an artificial synapse [43]. Furthermore, several studies on the conduction modulation linearity of PCMO memristors have been reported [5,24,44,45]. An artificial synapse consisting of two PCMO memristors was fabricated to improve learning accuracy [46]. Furthermore, the use of a peripheral circuit in the PCMO synapse device improved the conductance linearity and conductance ratio [24]. However, the fabrication of a high-density device using this PCMO memristor is difficult because this memristor needs an additional peripheral circuit. Therefore, enhancing the linearity of the conduction modulation of the PCMO memristor intended for application as an artificial synapse has become urgent. Furthermore, a systematic study of the fundamental synaptic properties of the PCMO memristor is also necessary.

In this study, amorphous PCMO films were grown on a TiN/SiO_2_/Si (TiN–Si) substrate at 300 °C. The PCMO memristors were designed to mimic several biological synaptic characteristics and were grown by varying the oxygen pressure (OP) to control the number of OVs in the PCMO film. The memristors fabricated in this manner were then used to investigate the effect of the number of OVs on the conduction modulation linearity. The PCMO film deposited at low OP contained a large number of OVs and exhibited linear conduction modulation. This suggests that conduction modulation linearity can be obtained by controlling the OP during the growth process. Moreover, this simple method, which was used in this study to improve the conduction modulation linearity, could also be applied to other RRAM memristors whose switching behaviors are determined by the growth of OV filaments. In addition, simulation based on a convolutional neural network (CNN) showed that a PCMO memristor with good conduction modulation linearity exhibited increased classification accuracy. Moreover, the PCMO device developed in this study has a thin PCMO film (30–40 nm) with a simple device structure. However, a thick PCMO film (~500 nm) or complicated device structures has been used for PCMO RRAM devices [47,48,49]. Therefore, the PCMO RRAM device developed in this study can be beneficial for fabricating high-density array circuits.

## 2. Experimental Procedures

PCMO thin films were deposited on the TiN–Si substrates at 300 °C and at 100, 200, and 300 mTorr OP using the pulsed laser deposition (PLD) method. The thickness of the PCMO films was approximately 30–40 nm. A PCMO ceramic target with a diameter of 1.0 in was sintered at 1200 °C. The wavelength, repetition rate, and energy fluency of the Nd-YAG laser beam (EKSPLA NL303HT, Vilnius, Lithuania) were 266 nm, 10 Hz, and 4 J/cm^2^, respectively. This laser beam was focused on the PCMO target, which was rotated inside a vacuum chamber at various OP. Pt was deposited on the PCMO film by DC sputtering (Korea vacuum tech KVS101, Seoul, Korea) to form the top electrode. The structural characteristics of the PCMO films were studied by X-ray diffraction (XRD; Rigaku D/max–RC, Tokyo, Japan) and field-emission scanning electron microscopy (FE-SEM; Hitachi S-4300, Tokyo, Japan). The chemical binding energies of the O1s and N1s orbitals were measured by XPS (ULVAC-PHI X-tool, Kanagawa, Japan). The current versus voltage (*I–V*) curves, DC-sweep endurance, and retention characteristics of the PCMO memristor were recorded using a source meter (Keithley 2400, Solon, OH, USA). Current compliance was utilized to limit the current flow to prevent complete dielectric breakdown of the PCMO memristors. The synaptic properties, such as the nonlinear transition properties and STP–LTP transformation, were measured using a semiconductor characterization system (Keithley 4155C SCS, Solon, OH, USA) and pulse function arbitrary noise generator (Agilent 81110A, Santa Clara, CA, USA). A semiconductor characterization system (Keithley 4200 SCS, Solon, OH, USA) with pulse measurement units (PMUs) was used to obtain the SRDP and STDP characteristics of PCMO memristors. The measured device properties were used to conduct pattern recognition simulation with the aid of a convolutional neural network (CNN). Images of 10,000 handwritten digits supplied by MNIST were used for the simulation [50]. Five-fold cross-validation was used to confirm the reliability of the simulation results of the CNN. Details of the CNN simulation process are provided in Supplementary Information 7.

## 3. Results and Discussion

**Figure 1:**
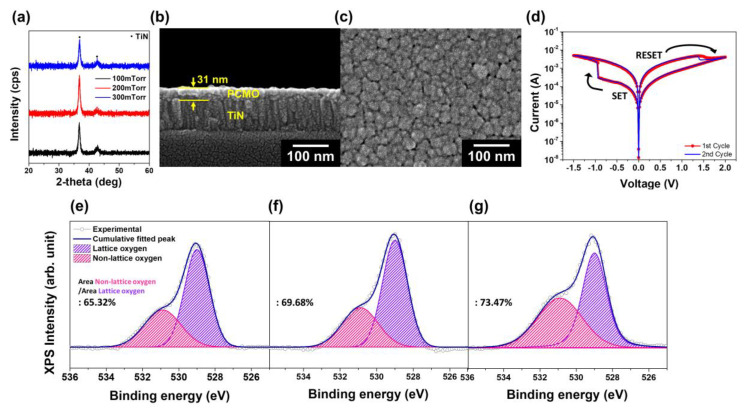
(**a**) XRD patterns of the PCMO thin films deposited on the TiN–Si substrate at 300 °C by varying the OP. SEM images of the (**b**) cross-section and (**c**) surface of the PCMO thin film deposited at 100 mTorr OP. (**d**) *I–V* curve of the PCMO thin film deposited at 100 mTorr OP. XPS O1s spectra of the PCMO films grown by varying the OP: (**e**) 300 mTorr, (**f**) 200 mTorr, and (**g**) 100 mTorr.

Figure 1a shows the grazing incidence XRD patterns of the PCMO thin films deposited on the TiN–Si substrate at 300 °C by varying the OP and with a grazing incidence angle of 3° [51]. The peaks of the crystalline PCMO phase were not detected, suggesting that these films consisted of the amorphous PCMO phase. A cross-sectional SEM image of the PCMO thin film, deposited at 100 mTorr OP, is shown in Figure 1b. The PCMO film with a thickness of 31 nm was well developed on the TiN electrode. Similar results were also observed for the PCMO thin films deposited at different levels of OP (Figure S1a,b). The surface of the PCMO film deposited at 100 mTorr OP is shown in Figure 1c, which shows that a dense microstructure developed in this PCMO film. The other PCMO thin films deposited by varying the OP yielded similar results (Figure S1c,d). The *I–V* curve of the PCMO thin film deposited at 100 mTorr OP is shown in Figure 1d, and this PCMO film showed a normal bipolar switching curve: a set voltage of −0.9 V, and a reset voltage of 1.6 V. A forming procedure was not required to obtain this *I–V* curve, and the *I–V* curve of the second sweep was similar to the first *I–V* curve, confirming that the *I–V* curves could be obtained without the forming process for the PCMO film deposited at 100 mTorr OP. The PCMO film deposited at 200 mTorr OP also displayed a normal bipolar switching curve without a forming process (Figure S2a). However, this PCMO film showed a higher set voltage of approximately −2 V and high resistance in the high resistance state (HRS), as indicated in Figure S2a, probably owing to a decrease in the number of OVs in the film.

The number of OVs in the PCMO film was investigated by conducting XPS O1s analysis of the PCMO films grown at different OP, as shown in Figure 1e–g. The spectra were observed to exhibit two peaks: the peak at 529 eV was attributed to the oxygen ions occupying lattice sites, and that at 531 eV corresponded to oxygen ions in non-lattice sites [52,53]. The occupation of a non-lattice site by an oxygen ion resulted in the formation of an OV in the lattice site. This suggests that the number of OVs increased with increasing intensity of the peak at 531 eV. For the PCMO film deposited at 300 mTorr OP, the intensity of the peak at 531 eV was approximately 65.3% of the total intensity of all the peaks (Figure 1e), and the intensity of this peak increased with decreasing OP, as shown in Figure 1f,g. In the case of the PCMO film grown at 100 mTorr OP, the intensity of the peak at 531 eV was approximately 73.5% of the total peak intensity, indicating that many OVs were formed in this PCMO film. According to the XPS results, the number of OVs in the PCMO film decreased with increasing OP during the deposition of the film (Figure 1e–g), and the PCMO film deposited at a high OP of 300 mTorr contained a small number of OVs (Figure 1g). Hence, this PCMO thin film required a forming procedure to obtain the *I–V* curve (Figure S2b). Hence, it can be suggested that the bipolar switching properties of these amorphous PCMO films are related to the number of OVs in the film. In addition, bipolar switching properties were not observed in the PCMO films deposited at 5 mTorr and 50 mTorr OP (Figure S2c). Furthermore, the current level increased with a decrease in OP due to the large number of OVs present in these PCMO films.

**Figure 2:**
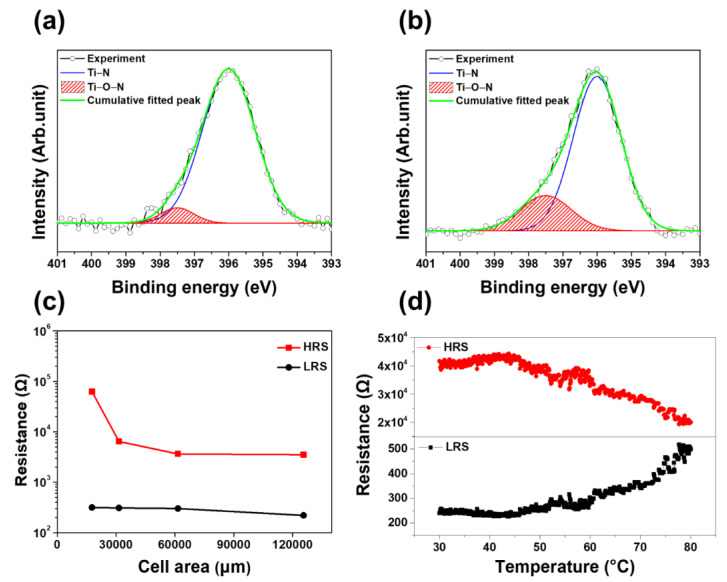
XPS N1s spectra of the PCMO film in the (**a**) HRS and (**b**) LRS. (**c**) Variation in the *R*_HRS_ and *R*_LRS_ values with respect to the size of the top electrode and (**d**) change in the *R*_HRS_ and *R*_LRS_ values with respect to the temperature. The PCMO film was grown at 100 mTorr OP.

The PCMO film deposited at 100 mTorr OP was analyzed at the PCMO/TiN interface by using XPS to investigate the effect of the OVs on the switching properties of the PCMO film. Figure 2a,b display the spectra of XPS N1s obtained from this PCMO film in the high-resistance state (HRS) and low-resistance state (LRS), respectively. A high-intensity Ti–N peak, originating from the TiN electrode, was observed at 396 eV for the PCMO film in the HRS (Figure 2a). Moreover, a low-intensity peak corresponding to the Ti–O–N bonding was observed at 397.5 eV. This peak resulted from the bonding between the oxygen ions and the TiN, indicating the presence of a very small number of oxygen ions in the TiN electrode in the HRS. However, the XPS analysis of the PCMO film in the LRS showed an intense Ti–O–N peak (Figure 2b), implying that a large number of oxygen ions migrated into the TiN bottom electrode during the set process. Moreover, the OVs, which were generated in the PCMO film during the set process, were considered to form the conducting OV filaments in the PCMO film. The PCMO films formed at 200 and 300 mTorr OP exhibited similar results (Figure S2c,d). Figure 2c displays the variation in the resistance of the PCMO film deposited at 100 mTorr OP in the HRS (*R*_HRS_) and LRS (*R*_LRS_) with respect to the size of the top electrode of the PCMO memristor. The value of *R*_HRS_ decreased as the size of the top electrode increased, suggesting that this current might be explained by a mechanism related to the interface of the electrode/PCMO film or the PCMO film [39]. On the contrary, the resistance of the PCMO film in the LRS did not change as the size of the top electrode increased (Figure 2c). Therefore, the current in the PCMO in the LRS can be explained by the presence of conducting OV filaments formed during the set process. Furthermore, the currents in the PCMO memristors in both the LRS and HRS can be explained by Ohmic conduction and space-charged-limited-current (SCLC), respectively, as displayed in Figure S3a–c. The SCLC mechanism was previously used to explain the current of the HRS of the PCMO film, although the OVs behaved as trap sites in the PCMO film [54]. Figure 2d exhibits the change in the resistance of the PCMO film in the HRS and LRS with respect to the measuring temperature. The resistance of the PCMO film in the HRS decreased with an increase in the temperature, indicating that this PCMO film has insulating properties [55]. These properties could be the result of the OV filaments possibly being disconnected during the reset process. However, the resistance of the PCMO film in the LRS increased with increasing temperature, implying that, in the LRS, the PCMO film has metallic conducting properties [55]. This result implies that the conducting OV filaments were connected in the LRS, inducing metallic properties in the PCMO film. Furthermore, the bipolar switching mechanism of the PCMO memristors fabricated in this study is attributed to the formation and disruption of the OV filaments. In addition, the PCMO films grown by varying the OP had excellent reliability characteristics, as shown in Figure S4a–c, indicating that these PCMO films could be utilized as a memristor.

**Figure 3:**
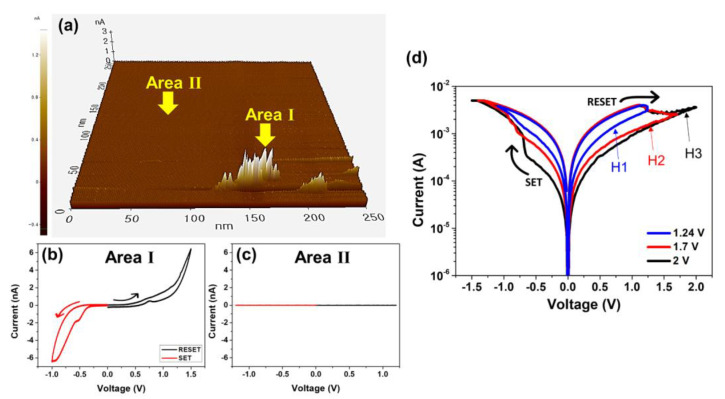
(**a**) Current map for the PCMO memristor in the LRS measured at 1.6 V. Bipolar switching curves obtained from (**b**) area I and (**c**) area II. (**d**) *I–V* plots of the Pt/PCMO/TiN–Si memristor measured at different reset voltages.

To verify the existence of the conducting OV filaments in the PCMO film in the LRS, the PCMO memristor in both the HRS and LRS was investigated using conductive atomic force microscopy (CAFM). Figure 3a exhibits a plot of the current in the PCMO memristor in the LRS obtained at 1.6 V, where the area of the PCMO film is 0.25 × 0.25 μm^2^. A current of approximately 8.5 nA was detected in area I, but current was not detected in area II (Figure 3a). The *I–V* curves were measured for areas I and II of the PCMO film (Figure 3a). A bipolar switching curve was measured for area I, as displayed in Figure 3b. However, current could not be measured for area II (Figure 3c). This suggests that the OV filaments were formed in area I of the PCMO film and behaved as a current path in the LRS. Moreover, the bipolar switching curve measured for area I of the PCMO memristor was attributable to the presence of the OV filaments in this area. Hence, it can be concluded that the switching behavior of the Pt/PCMO/TiN–Si memristor can be explained by the formation and rupture of the OV filaments in the PCMO film. Figure 3d shows the *I–V* plots of the Pt/PCMO/TiN memristor measured at different reset voltages (*V_RESET_*) to demonstrate the continuous change in the resistance level of the PCMO memristor. The PCMO film was deposited at 100 mTorr OP. The continuous change in the resistance is required for application as an artificial synapse [56]. Four resistance levels composed of one LRS and three HRSs were measured from the PCMO memristor. The three HRSs, namely *H_1_*, *H_2_*, and *H_3_*, were measured by using *V_RESET_* values of 1.25, 1.75, and 2 V, respectively, and these HRSs also had good reliability (Figure S4d). Furthermore, additional HRSs are considered likely to be detected by further changing the *V_RESET_* values. These results confirm that the PCMO memristor exhibits a continuous change in its resistance levels, indicating that the PCMO memristor could be employed as an artificial synapse with several synaptic weights.

**Figure 4:**
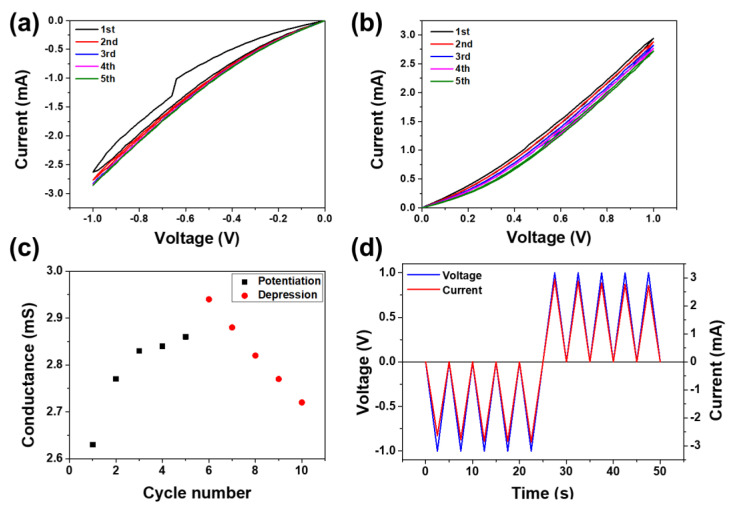
*I–V* plots of the PCMO film measured by five successive applications of (**a**) negative voltage varying from 0 to −1.0 V and (**b**) positive voltage varying from 0 to 1.0 V. (**c**) Variations in the conductance of the PCMO memristor with respect to the number of sweeps and (**d**) the current and voltage with respect to time.

The artificial synaptic properties of the Pt/PCMO/TiN–Si memristor were also studied. Because the nonlinear transmission property is an important biological synaptic property, it has been realized in the PCMO memristor. Figure 4a displays the *I–V* plots of the PCMO film grown at 100 mTorr OP as five negative voltage sweeps (0 to −1.0 V) were supplied to the PCMO memristor. The current in the PCMO memristor was negatively enhanced with the application of each voltage sweep. However, when five positive voltage sweeps (0 to 1.0 V) were supplied to the PCMO memristor, the current decreased with the supply of each voltage sweep, as displayed in Figure 4b. The conductance obtained at 0.1 V is displayed as a function of the number of sweeps (Figure 4c) to enable the variation in the current (or conduction) to be clearly visualized. In addition, the voltage and current curves were obtained as a function of time (Figure 4d). These results clearly indicate that the current in the PCMO memristor changed with the supply of successive DC biases to the device. Similar results were obtained from the PCMO films deposited by varying the OP, as shown in Figure S5a–d. Because the conduction (or current) of the PCMO memristor can be considered as the synaptic weight, these continuous variations in conductance with the application of repeated DC biases are regarded to be representative of the nonlinear transmission property in a biological synapse. The PCMO memristors can therefore be concluded to emulate the nonlinear transmission property of a biological synapse. 

**Figure 5:**
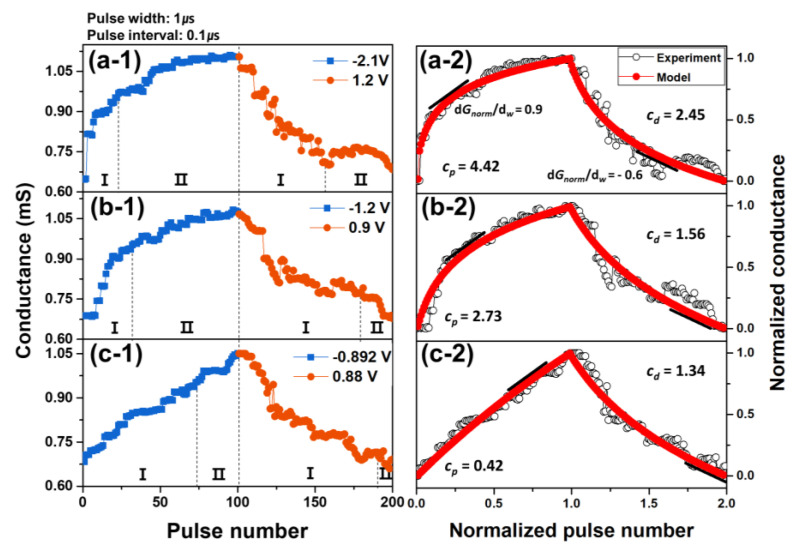
Variation of conductance with the applications of 100 P-spikes and 100 D-spikes to the PCMO memristor grown under various Ops: (**a-1**) 300 mTorr, (**b-1**) 200 mTorr, and (**c-1**) 100 mTorr. Potentiation and depression curves with *c_p_* and *c_d_* values for the PCMO memristor grown under various Ops: (**a-2**) 300 mTorr, (**b-2**) 200 mTorr, and (**c-2**) 100 mTorr.

Figure 5(a-1–c-1) displays the variation in the conductance with the successive application of 100 negative pulses (potentiation spikes (P-spikes)) and 100 positive pulses (depression spikes (D-spikes)) to the PCMO memristors fabricated by varying the OP. The magnitudes of the P-spike and D-spike, which were supplied to each memristor, are indicated in each figure. The potentiation curve was composed of two regions, i.e., part I and part II. The rate at which the conductance increases was large in part I and became small in part II. The depression curve displayed the identical tendency: the conductance decreased rapidly in part I and decreased slowly in part II. Part I of the PCMO film grown at 300 mTorr OP was narrow, whereas part II was wide (Figure 5(a-1)). The size of part I increased but that of part II decreased as the OP decreased, as shown in Figure 5(b-1,c-1). Moreover, the linearity of the conduction modulation of the PCMO memristor increased with decreasing OP. This linearity of the PCMO memristor was required because the learning (or writing) accuracy was improved as the linearity of the conduction modulation of RRAM increased [18]. The curvatures of the potentiation (*c_p_*) and depression (*c_d_*) curves of the PCMO memristors grown by varying the OP were simulated by using theoretical models, as shown in Supporting Information 6. The calculated curves and their curvatures are also shown in Figure 5(a-2–c-2). The linearity of the curve was enhanced when the curvature of the curve approached zero. The *c_p_* and *c_d_* values of the PCMO film grown at 300 mTorr OP were 4.42 and 2.45, respectively (Figure 5(a-2)) and decreased with decreasing OP. The PCMO film deposited at 100 OP exhibited the smallest *c_p_* and *c_d_* values of 0.42 and 1.34, respectively, as shown in Figure 5(c-2). Therefore, the PCMO film grown at low OP (100 mTorr) can be concluded to display good conduction modulation linearity. Additionally, the slope of the normalized conductance curve was used to determine the division between parts I and II, and the slope is shown in Figure 5a–c.

**Figure 6:**
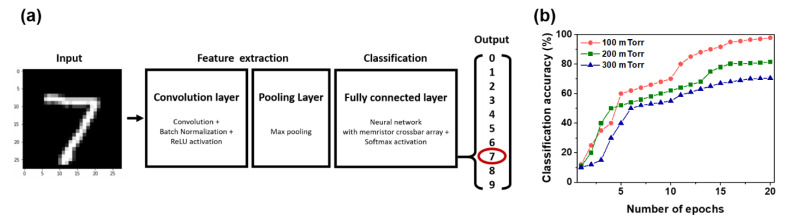
(**a**) Schematic diagram of the CNN structure and (**b**) calculated recognition accuracy of the MNIST patterns as a function of the number of training epochs for the PCMO memristors grown by varying the OP.

The classification accuracy is generally accepted to be related to the conduction modulation linearity [25]. Hence, the PCMO memristor, which was fabricated using the PCMO film grown at 100 mTorr OP, was expected to have improved classification accuracy. The classification accuracy of the PCMO memristors grown by varying the OP was simulated using a CNN. The CNN model consisted of two parts, namely feature extraction and classification, as shown in Figure 6a. Feature extraction was conducted using input, convolution, and pooling layers, and the features of input images were extracted, as shown in Figure 6a. Classification was carried out by using a fully connected layer and an output layer, and the input image was classified using a neural network (Figure 6a). Details of the CNN are described in Supporting Information 7. Figure 6b shows the change in the classification accuracy of the PCMO memristors with respect to the number of epochs when the MNIST patterns were used as input. The accuracy of the PCMO memristors was enhanced and eventually saturated as the number of epochs increased. The maximum accuracy of the digit recognition for the PCMO memristor grown at 300 mTorr OP was approximately 70%, and it increased for PCMO memristors fabricated at low OP (Figure 6b). In particular, the PCMO memristor fabricated at 100 mTorr OP, which showed the best conduction modulation linearity, exhibited the highest classification accuracy of 95.85%, as shown in Figure 6b. Similar results were obtained when 5-fold cross-validation was used, as listed in Table S1. Therefore, the classification accuracy of an artificial synapse could be concluded to improve as the conduction modulation linearity increases. In addition, the PCMO film grown at 100 mTorr OP also exhibited reliable multistate retention of the transmission curve, as shown in Figure S6c.

**Figure 7:**
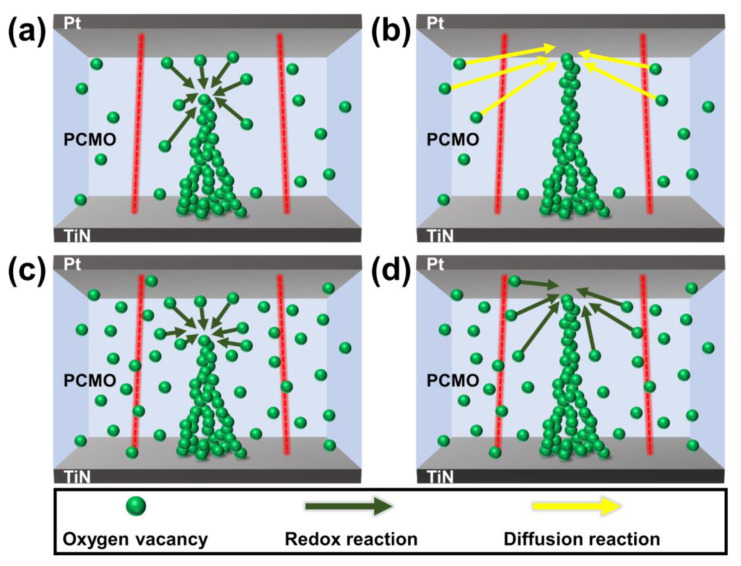
Schematic diagrams showing (**a**) the amount of OVs and (**b**) growth of the filament in the PCMO film grown at 300 mTorr; (**c**) the amount of OVs and (**d**) the growth of the filament in the PCMO film grown at 100 mTorr.

Because the switching properties of the PCMO memristor are related to the OV filaments, the change in the conductance of the PCMO memristor can be described by the change in the size of the OV filaments. The conduction range of the potentiation and depression curves corresponds to the HRS in the PCMO films. Hence, the variation in the conductance in the potentiation (or depression) curve can be described by the change in the length of the OV filaments in the PCMO film. According to previous studies, the growth (or shrinkage) of the OV filament could be explained by two reactions: the redox reaction (fast reaction) and oxygen ion diffusion (slow reaction) [18,26,29,30]. Parts I and II in the potentiation (or depression) curve were described by the redox reaction and oxygen diffusion process, respectively [18]. Moreover, it has been suggested that the linearity of the conduction modulation can be enhanced when the change in the size of the filament is mainly determined by one of these processes [18,26,29,30]. A small number of OVs existed for the PCMO film grown at 300 mTorr OP, as shown in Figure 7a. When P-spikes were applied to the PCMO memristor, OVs near the OV filament joined the OV filament through the redox process, triggering an abrupt increase in the current (part I in Figure 5a). However, these OVs were soon depleted as only a small number of OVs existed near the OV filament. Hence, the OVs further from the filament diffused into the filament and contributed to the increase in the filament size (Figure 7b). In this instance, the growth of the filament was controlled by the diffusion of OVs, which slowly increased the current (part II in Figure 5a). Therefore, the growth of the OV filaments in the PCMO film grown at 300 mTorr is controlled by two mechanisms, redox reaction and OV diffusion process, leading to nonlinear conduction modulation in the potentiation curve, as shown in Figure 5a. The shrinkage of the filaments in the PCMO film grown at 300 mTorr OP is also controlled by two mechanisms. Hence, the nonlinear conduction modulation was also observed in the depression curve (Figure 5a).

However, for the PCMO film grown at 100 mTorr OP, numerous OVs existed in the PCMO film (Figure 7c), which contributed to the growth of the OV filament through the redox process, as shown in Figure 7d. Therefore, the growth of the OV filaments in the PCMO film grown at 100 mTorr is controlled by one mechanism, the redox reaction, resulting in linear conduction modulation, as shown in Figure 5c. Furthermore, since the shrinkage of the filaments in this PCMO film is controlled solely by the redox reaction, the depression curve also exhibits a linear conduction modulation (Figure 5c). This suggests that the redox reaction is the major growth mechanism driving the formation of OV filaments, thereby improving the conduction modulation linearity. Particularly, the size of part I in the potentiation (or depression) curve increased with decreasing OP, as shown in Figure 5a–c. This confirms the redox reaction to be the major growth mechanism underlying the formation of OV filaments. The improvement in the linearity of the conduction modulation in the PCMO film deposited at 100 mTorr OP can therefore be attributed to the presence of the large number of OVs in the PCMO film.

**Figure 8:**
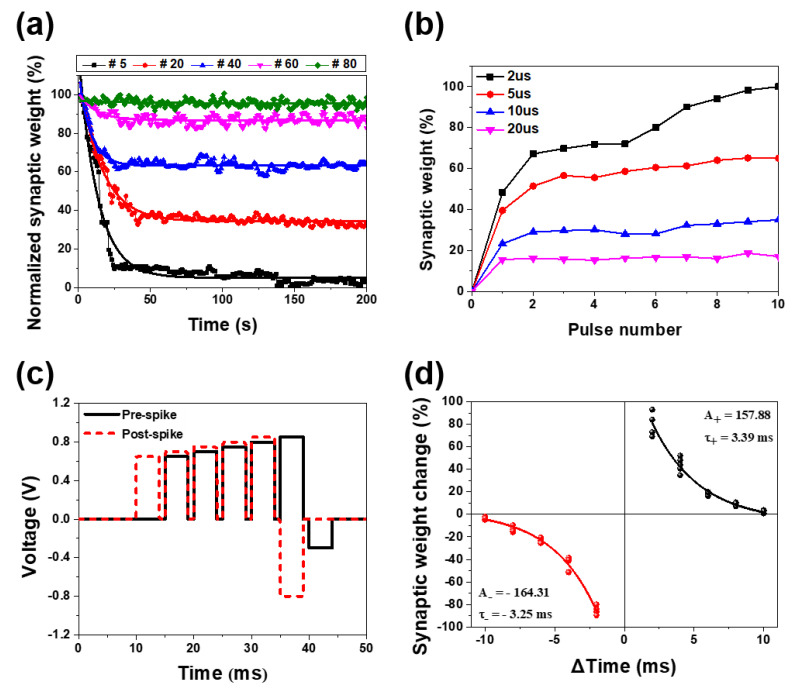
Variation in the synaptic weight as a function of (**a**) the retention time after the application of a different number of P-spikes, and (**b**) the number of pulses measured with P-spikes with different pulse intervals. (**c**) Pre- and post-spikes applied to PCMO memristor and (**d**) variation in Δ*w* with respect to Δ*t*.

The PCMO memristor fabricated at 100 mTorr OP is considered to be an effective artificial synapse because this memristor is characterized by linear conduction modulation and high classification accuracy. This encouraged us to additionally study various biological synaptic characteristics of this memristor. Because the STP–LTP transformation emulates the memory process of the human brain, it is an essential synaptic characteristic of an artificial synapse. Hence, the STP–LTP transformation of the PCMO memristor was examined, as shown in Figure 8a, which shows the variation in the synaptic weight with respect to the retention time after varying the number of P-spikes. When five P-spikes were used, the amount of LTP, which was transmitted from the STP, was small at approximately 10% of the STP. The LTP was enhanced when the number of P-spikes increased, i.e., 95% of the STP was transmitted to LTP when 80 P-spikes were supplied (Figure 8a). This indicates that an STP–LTP transformation occurred in the PCMO memristor. This transformation was in good agreement with the memory-loss property of the biological brain, as shown in Figure S8a,b. In addition, the change in the current accompanying the STP–LTP transformation ranged from 105 to 135 μA at 0.1 V (reading voltage). Therefore, the STP–LTP transformation in the PCMO memristor occurred in the HRS and could be attributed to the change in the length of the OV filament (Figure S8c–e). The SRDP characteristic of the PCMO memristor was also investigated, and Figure 8b exhibits the synaptic weight change with respect to the number of P-spikes, as measured using the number of P-spikes with different pulse intervals. The synaptic weight increased when the number of P-spikes increased. However, this increase in the synaptic weight could be negligibly small for large P-spike intervals of 20 μs. The increase in the synaptic weight with an increasing number of P-spikes increased as the P-spike interval decreased. The synaptic weight increased considerably with increasing P-spike number for a small P-spike interval of 2 μs (Figure 8b). These results indicate that the SRDP characteristic of a biological synapse was realized in the PCMO memristor. Likewise, the STDP characteristic of a biological synapse was also emulated in the PCMO memristor. The pre- and post-spikes are illustrated in Figure 8c, and the various net-spikes, which were used to obtain the synaptic weight change (Δ*w*), are shown in Figure S9a–d). The Pt top electrode and TiN bottom electrode in the Pt/PCMO/TiN memristor corresponded to the pre- and post-neutrons, respectively. Application of the spike to the Pt top electrode before the TiN bottom electrode resulted in the time difference between the pre- and post-spikes (Δ*t*) being positive. In contrast, application of the spike to the TiN bottom electrode before the Pt top electrode yielded negative Δ*t*. Figure 8d displays the variation in Δ*w* as a function of Δ*t* with the PCMO memristor potentiated as Δ*t* > 0 and depressed as Δ*t* < 0. The potentiation and depression were large when the absolute value of Δ*t* was small, and they were small when the absolute value of Δ*t* was large. This indicates that the STDP property of a biological synapse was realized in the PCMO memristor. The STDP characteristic of a biological synapse can be expressed by the following equation:

(1) $$\Delta W\;=\;{\{}_{C_{-}e^{-|\Delta t|/\tau_{-}}\;+\;\Delta W_{0-},\;\;\;\Delta t\;<\;0}^{C_{+}e^{-|\Delta t|/\tau_{+}}\;+\;\Delta W_{0+},\;\;\;\Delta t\;>\;0}$$

where *C*_+_ and *C*_−_ are constants, *τ*_+_ and *τ*_−_ are time constants, and Δ*W*_0+_ (or Δ*W*_0−_) is Δ*W* at infinite Δ*t* (or −Δ*t*). The STDP data of the PCMO memristor were well fitted by Equation (1), as shown in Figure 8d, and the values of *C*_+_/*C*_−_ and *τ*_+_/*τ*_−_ were determined as 157.9/−164.3 and 3.4 ms/3.3 ms, respectively. This result confirms that the STDP of a biological synapse is well emulated in the PCMO memristor. The results presented above show that the PCMO memristor can mimic several of the biological synaptic properties, and the memristor also exhibits good conduction modulation linearity. The PCMO memristor fabricated at low OP of 100 mTorr is therefore concluded to be a promising artificial synapse. In addition, the device size and HRS current of the PCMO film developed in this study are relatively large compared to those of the commercial device. However, the size and thickness of the PCMO film in PCMO devices can be readily reduced when produced by a semiconductor company, since the device has a very simple structure (Pt/PCMO/TiN–Si) and fabrication process, including a low growth temperature of 300 °C. Moreover, the linearity of the conduction modulation is readily achieved in a small PCMO device as it is obtained by controlling the OP during the growth of the PCMO film. Therefore, the miniaturization and conduction modulation linearity of the PCMO device are readily achieved.

## 4. Conclusions

A dense amorphous phase was developed in the PCMO film deposited on a TiN–Si substrate at 300 °C and at 100 mTorr OP using PLD. This PCMO film exhibited normal bipolar switching without the forming procedure. However, the PCMO film that was deposited at a high OP of 300 mTorr needs a forming procedure to show bipolar switching because this film contains a small number of OVs relative to that deposited at 100 mTorr OP. The switching characteristic of the Pt/PCMO/TiN memristor can be attributed to the establishment and disruption of the OV filaments. The PCMO memristor fabricated at 100 mTorr OP exhibited four resistance levels when the reset voltage was changed. This indicates that this PCMO memristor exhibits a continuous change in its resistance level, which is required for application as an artificial synapse. The current in the PCMO memristor decreased when negative voltage was successively applied, indicating that the nonlinear transmission properties of a biological synapse were replicated in the PCMO memristor. The conductance of PCMO memristors increased with the application of P-spikes and decreased with the application of D-spikes. A PCMO memristor would require linear conduction modulation for application to the neuromorphic computing system. The conduction modulation linearity increased as the number of OVs increased, because the change in the size of the OV filaments is mainly attributable to the redox reaction in film with a large number of OVs. The PCMO memristor fabricated at low OP of 100 mTorr contained a large number of OVs and exhibited good conduction modulation linearity. The results of the CNN simulation showed that the PCMO memristor fabricated at 100 mTorr OP, which has good conduction modulation linearity, exhibited higher pattern recognition accuracy compared to the other PCMO memristors. This result confirms that conduction modulation linearity considerably affects the classification performance of neuromorphic computing. Moreover, the PCMO memristor fabricated at 100 mTorr OP exhibited various properties of a biological synapse such as STP–LTP transformation, SRDP, and STDP.

## Supplementary Materials

The following are available online at https://www.mdpi.com/article/10.3390/nano11102684/s1, Figure S1: Cross-sectional SEM images of the PCMO films grown at OPs of (a) 200 mTorr and (b) 300 mTorr. SEM images of the surface of the PCMO thin film grown at OPs of (c) 200 mTorr and (d) 300 mTorr, Figure S2: *I-V* curves of the PCMO films grown at (a) 200 mTorr, (b) 300 mTorr OP and (c) 50 and 5 mTorr. XPS N1s spectra in the (d-1) HRS and (d-2) LRS for the PCMO film grown at 200 mTorr OP. XPS N1s spectra in the (e-1) HRS and (e-2) LRS for the PCMO film grown at 300 mTorr OP, Figure S3: *I-V* curves in HRS and LRS for the PCMO films grown by varying the OP: (a) 100 mTorr, (b) 200 mTorr, and (c) 300 mTorr, Figure S4: Currents in the LRS and HRS measured up to 300 DC cycles for the PCMO films grown by varying the OP: (a-1) 100 mTorr, (b-1) 200 mTorr, and (c-1) 300 mTorr. Retention characteristics of the LRS and HRS measured up to 10^3^ s for the PCMO memristors grown by varying the OP: (a-2) 100 mTorr, (b-2) 200 mTorr, and (c-2) 300 mTorr. (d) Retention characteristics of LRS, H_1_, H_2_, and H_3_, measured up to 10^3^ s for the PCMO memristor fabricated at 100 mTorr OP, Figure S5: *I-V* curves of the PCMO film grown at 200 mTorr OP measured with the application of five successive (a) negative voltage sweeps varying from 0 to −0.6 V and (b) positive voltage sweeps varying from 0 to 1.2 V. *I-V* curves of the PCMO film grown at 300 mTorr OP measured with the application of five successive (c) negative voltage sweeps varying from 0 to −1.5 V and (d) positive voltage sweeps varying from 0 to 1.1 V, Figure S6: (a) Various parameters of Equations (S1) and (S2) such as *G_max_*, *G_min_*, *c_p_*, *c_d_*, *β_p_*, and *β_d_* are indicated on the potentiation and depression curves. (b) Potentiation and depression curves with various *c_p_* and *c_d_* values. (c) Multistate retention of the PCMO film grown at 100 mTorr obtained from the transmission curve, Figure S7: (a) Schematic diagram of the basic convolutional neural network (CNN) architecture. (b) Diagram of neural networks in the form of crossbar array architecture with memristors, Figure S8: Schematics of the filament length in the PCMO memristor in (a) HRS, (b-1) with the application of five P-spikes, (b-2) after removal of the P-spikes, (c-1) with the application of 80 P-spikes, and (c-2) after removal of the P-spikes, Figure S9: (a) Pre-, (b) post-, and (c) various net-spikes applied to the PCMO memristors to obtain the synaptic weight change (Δ*w*), Table S1: Five-fold cross validation. Comparison of classification accuracies computed by CNNs with weights composed of conductance obtained at 100, 200, and 300 mTorr.
