# Supplementary material for: Effect of Oxygen Vacancy on the Conduction Modulation Linearity and Classification Accuracy of Pr0.7Ca0.3MnO3 Memristor
Source: Nanomaterials (Basel). 2021 Oct 12;11(10):2684. doi: 10.3390/nano11102684 (PMC8538184; doi:10.3390/nano11102684)
Supplement: Supplementary file 1 [file nanomaterials-11-02684-s001.zip › nanomaterials-1387858-supplementary.pdf]

nanomaterials

Effect of Oxygen Vacancy on the Conduction Modulation Linearity and Classification Accuracy of $Pr_{0.7}Ca_{0.3}MnO_3$ Memristor

Yeon Pyo [1,†], Jong-Un Woo [2,†], Hyun-Gyu Hwang [2], Sahn Nahm [2,3,*] and Jichai Jeong [1,*]

1 Department of Brain and Cognitive Engineering, Korea University, 145 Anam-ro, Seongbuk-gu, Seoul 02841, Korea. pyoyeon@korea.ac.kr
2 KU-KIST Graduate School of Converging Science and Technology, Korea University, 145 Anam-ro, Seongbuk-gu, Seoul 02841, Korea. jong41187@korea.ac.kr (J.-U.W.); hghwang263@korea.ac.kr (H.-G.H.)
3 Department of Materials Science and Engineering, Korea University, 145 Anam-ro, Seongbuk-gu, Seoul 02841, Korea.
* Correspondence: snahm@korea.ac.kr (S.H.); jcj@korea.ac.kr (J.J.)
† These authors contributed equally to this work.

1. SEM images of the PCMO films grown by varying the oxygen pressure

Figure S1a and S1b show the cross-sectional SEM images of PCMO films grown on TiN-Si substrates at 300 °C and at OPs of 200 and 300 mTorr, respectively. These PCMO films were well formed under these conditions with thicknesses ranging from 32 to 36 nm. The SEM images of the surfaces of these PCMO films are displayed in Figs. S1c and d and show that the films exhibit a dense microstructure. Therefore, it can be concluded that the amorphous PCMO films were well formed on the TiN-Si substrates at 300 °C by varying the OP.

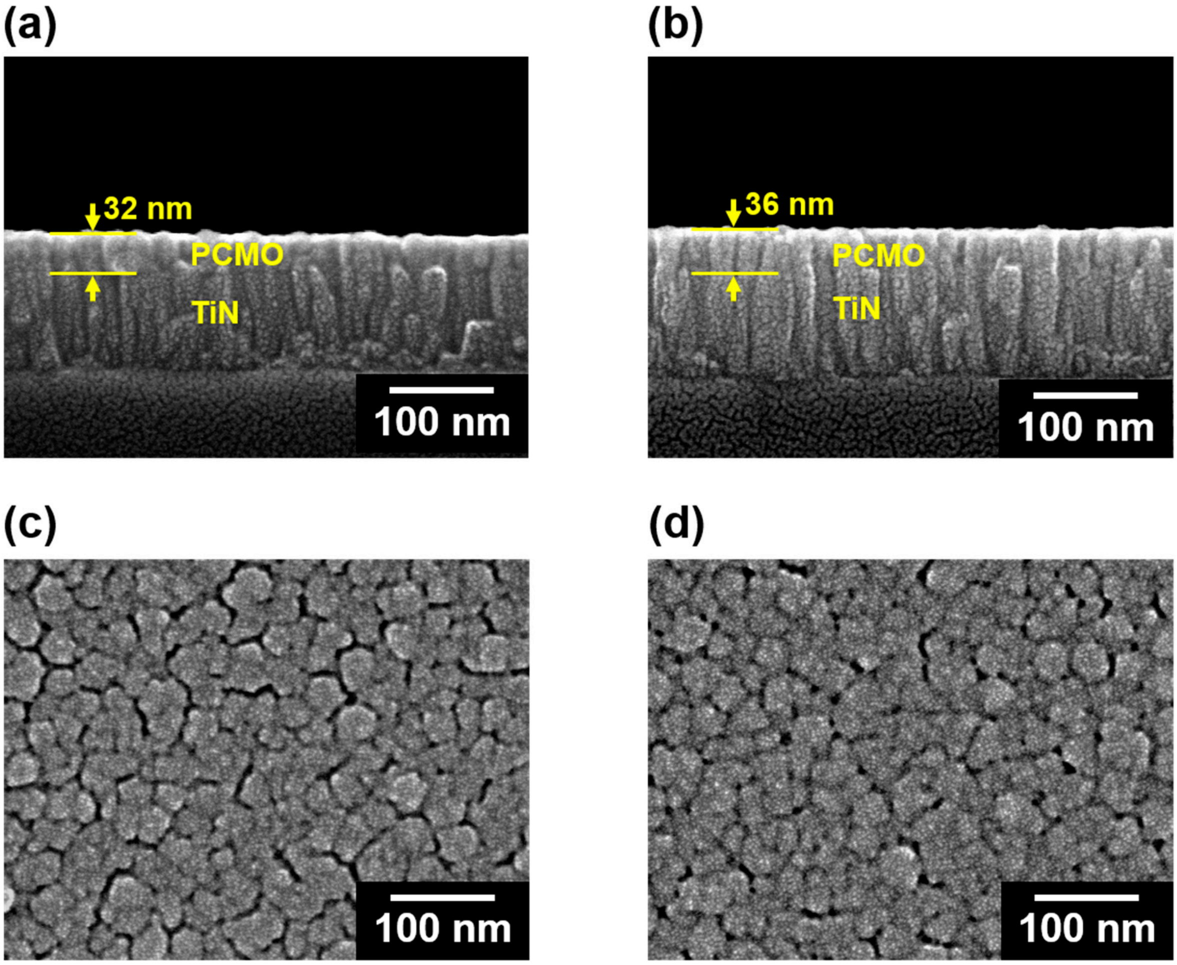

Figure S1. Cross-sectional SEM images of the PCMO films grown at OPs of (**a**) 200 mTorr and (**b**) 300 mTorr. SEM images of the surface of the PCMO thin film grown at OPs of (**c**) 200 mTorr and (**d**) 300 mTorr.

2. *I-V* curves and XPS results of the PCMO thin film grown by varying the OP

The *I-V* curve of the PCMO thin film grown at 200 mTorr OP is displayed in Figure S2a. A forming process was not needed to obtain this curve. The *I-V* curve of the second sweep is almost the same as the first *I-V* curve, confirming that the *I-V* curves can be obtained without the forming process for the PCMO film deposited at 200 mTorr OP. This PCMO film exhibited a typical bipolar switching curve and the set and reset voltages were –2 V and 2.5 V, respectively. This PCMO film had a large set voltage of –2 V and its resistivity in the HRS was high compared to that of the PCMO film grown at 100 mTorr OP. The number of oxygen vacancies (OVs) in this PCMO film could be smaller than those in the PCMO film grown at 100 mTorr OP because it was grown at higher OP. Therefore, the increased set voltage and resistance in the HRS of the PCMO film grown at 200 mTorr OP can be explained by the decreased number of OVs. Figure S2b shows the *I-V* curve of the PCMO film grown at high OP of 300 mTorr. A forming process is required to obtain the bipolar switching curve, confirming that the number of OVs decreased for the PCMO film grown at high OP. After the forming process, a typical bipolar switching curve with set voltage of –1.5 V and reset voltage of 2.5 V was obtained (Figure S2b). In addition, Figure S2c shows the *I-V* curves of the PCMO films deposited at 50 mTorr and 5 mTorr OP; however, bipolar switching properties were not observed in these thin films. Furthermore, the current level increased with decreasing OP due to the large number of OVs in the films grown at low OP.

XPS analysis was conducted on the PCMO films grown at OP of 200 mTorr and 300 mTorr to determine the effect of the OVs on the switching properties of these PCMO films. Figure S2(d-1) and (d-2) display the XPS N1s spectra of the PCMO film in the HRS and LRS, respectively, and this PCMO film was grown at 200 mTorr OP. The XPS analysis was conducted at the interface between the PCMO film and TiN electrode. The XPS result of the PCMO film in the HRS displays an intense Ti-N peak at 396 eV (Figure S2(d-1)) and could have originated from the TiN electrode. However, a very low-intensity peak is observed at 397.5 eV, which corresponds to Ti-O-N bonding owing to the bonding between the oxygen ions and the TiN electrode. This suggests that a very small number of oxygen ions existed in the TiN electrode in the HRS. However, the XPS result obtained from this PCMO film in the LRS exhibits an intense Ti-O-N peak (Figure 2(d-2)), implying that a large number of oxygen ions existed in the TiN electrode in the LRS and could have migrated to the TiN electrode during the set process. Moreover, the OVs could have been produced in this PCMO film during the set process, and they formed the conducting oxygen filaments in this PCMO film. Similar results were observed for the PCMO film grown at 300 mTorr OP, as shown in Figure S2(e-1) and (e-2). These results imply that the switching properties of the PCMO memristors formed in this study can be explained by the formation and rupture of the OV filaments.

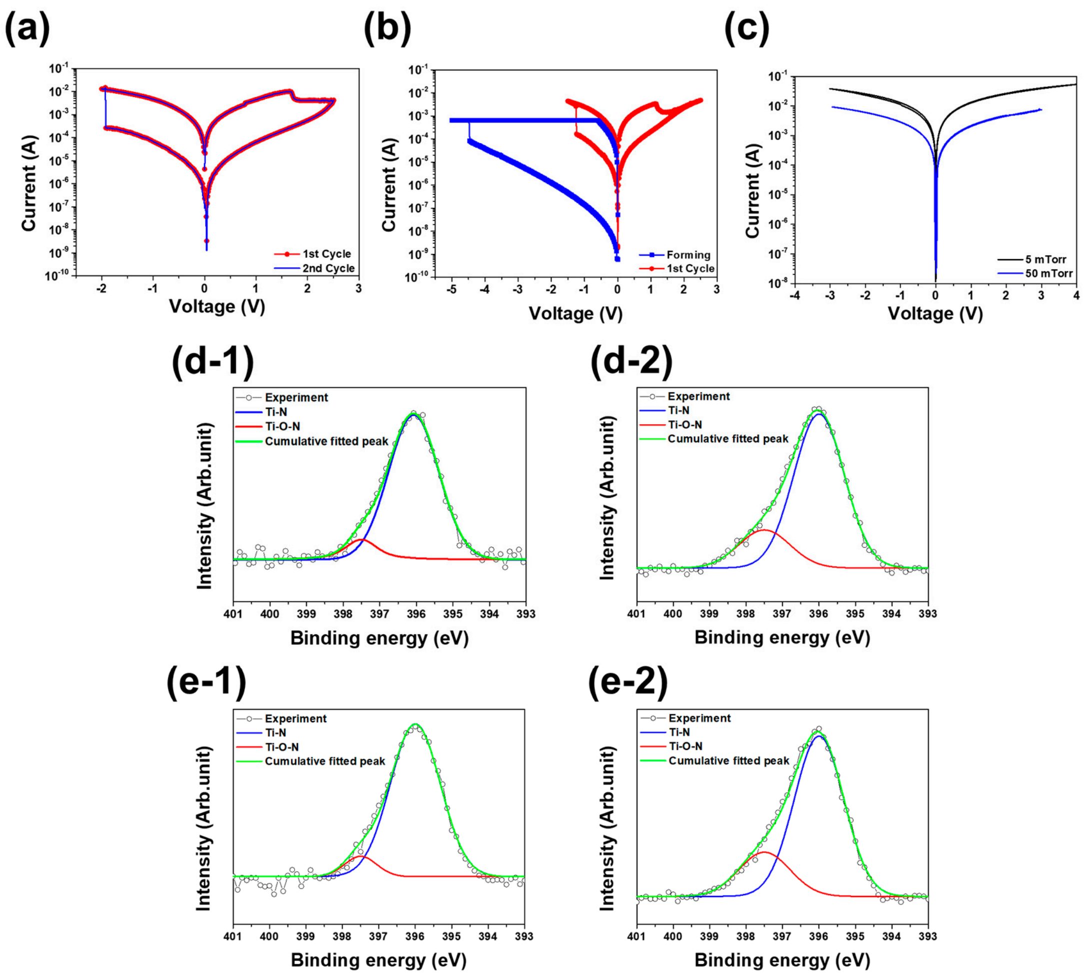

Figure S2. *I-V* curves of the PCMO films grown at (**a**) 200 mTorr, (**b**) 300 mTorr OP and (**c**) 50 and 5 mTorr. XPS N1s spectra in the (**d-1**) HRS and (**d-2**) LRS for the PCMO film grown at 200 mTorr OP. XPS N1s spectra in the (**e-1**) HRS and (**e-2**) LRS for the PCMO film grown at 300 mTorr OP.

3. Conduction mechanism of PCMO thin films grown by varying the OP

Figure S3a shows the *I-V* curves in the HRS and LRS for the PCMO film grown at 100 mTorr OP. The slope of the *I-V* curve of this PCMO film in HRS is 1.06 at low voltage, but it changes to 2.09 when the voltage exceeds 1.0 V. Therefore, it can be concluded that space-charge-limited conduction (SCLC) is the conduction mechanism of this PCMO film in the HRS [1]. In contrast, the slope of the *I-V* curve of this PCMO film in the LRS is 1.09, which is very close to 1.0. Hence, in the LRS, the PCMO film has metallic conductive properties, and Ohmic conduction is the current conduction mechanism of this PCMO film in LRS. The *I-V* curves in the HRS and LRS for the PCMO film grown at 200 mTorr OP are displayed in Figure S3b. The slope of the *I-V* curve of this PCMO film in the HRS is 1.1 at low voltage, and it changes to 2.06 when the applied voltage exceeds 0.4 V. Finally, the slope of the *I-V* curve changes to 7.85 when the applied voltage exceeds 2 V, indicating that SCLC is the conducting mechanism of this PCMO film in the HRS. On the contrary, the slope of the *I-V* curve of this PCMO film in the LRS is 1.06, which closely approximates 1.0. Hence, in the LRS, Ohmic conduction is the current conduction mechanism of this PCMO film. The PCMO film grown at 300 mTorr OP exhibits similar results, as shown in Figure S3c. Therefore, SCLC and Ohmic conduction are considered to be the current conduction mechanisms of this PCMO film in the HRS and LRS, respectively.

The SCLC mechanism requires an Ohmic contact between the electrode and film at the interface [2]. Therefore, it is understood that the OVs, which existed in the PCMO film

near the PCMO/TiN interface, decreased the potential barrier of this interface, resulting in a quasi-conductive interface between the TiN electrode and PCMO film. Furthermore, because the oxygen ions moved into the TiN electrode during the set process, the OVs in the PCMO film produced metallic conducting filaments in the PCMO film in the LRS. Therefore, Ohmic conduction is the current conduction mechanism of the PCMO film in the LRS.

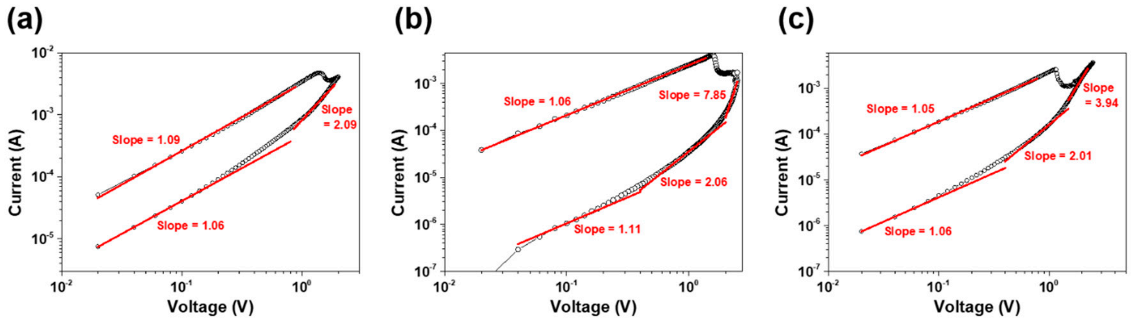

Figure S3. *I-V* curves in HRS and LRS for the PCMO films grown by varying the OP: (**a**) 100 mTorr, (**b**) 200 mTorr, and (**c**) 300 mTorr.

4. Reliability characteristics of PCMO thin films grown by varying the OP

The reliability properties of the PCMO films, which were grown on the TiN-Si substrate at 300 °C by varying the OP, were investigated. Figure S4(a-1) shows the currents in the LRS and HRS that are measured at 0.1 V after DC cycling from 2.0 to −1.5 V for the PCMO film grown at 100 mTorr OP. The LRS and HRS were well maintained after 300 cycles. Therefore, the PCMO ReRAM memristor that is fabricated at 300 °C and at OP of 100 mTorr exhibits a uniform distribution of R_{HRS} and R_{LRS}. Similar results were observed for the PCMO films grown at OP of 200 mTorr and 300 mTorr, as shown in Figure S4(b-1) and (c-1), respectively.

Figure S4(a-2) displays the retention properties of the LRS and HRS of the PCMO film grown at 100 mTorr OP, and it is clear that the R_{LRS} and R_{HRS} values remain constant up to 10^3 s. Therefore, the Pt/PCMO/TiN-Si ReRAM memristor with the PCMO film that was grown at 300 °C at 100 mTorr OP has good reliability characteristics. Similar results were observed for the PCMO films grown at 200 mTorr and 300 mTorr OP, as displayed in Figure S4(b-2) and (c-2). These results clearly show that the PCMO memristors can be used as ReRAM memristors. The retention properties of the PCMO memristor in the LRS and various HRSs were also investigated, as shown in Figure S4d. This PCMO memristor was fabricated at 100 mTorr OP. All states exhibited constant resistance up to 10^3 s. This suggests that this PCMO memristor has stable electrical reliability properties for an artificial synapse.

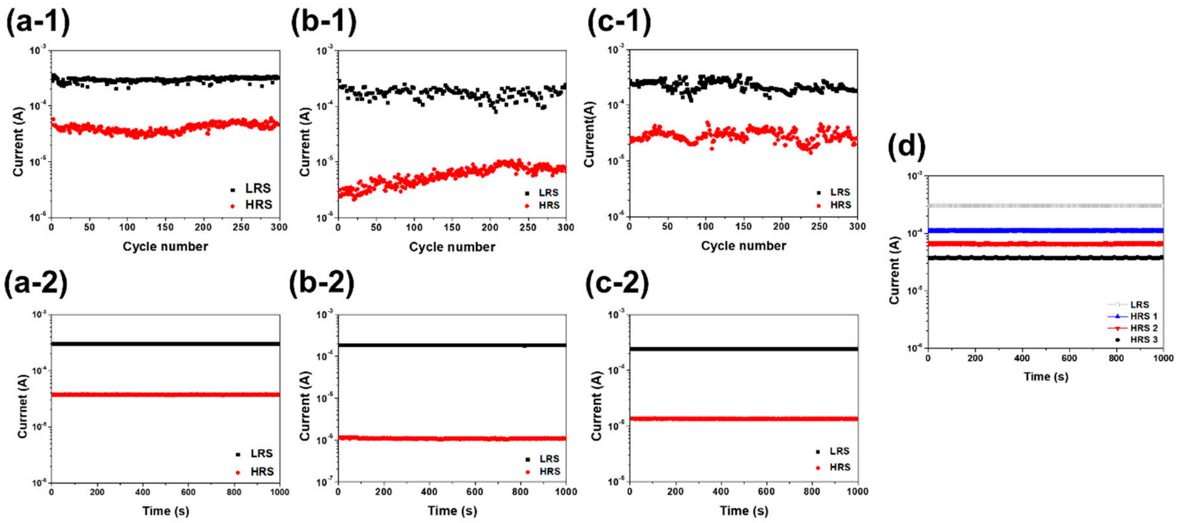

Figure S4. Currents in the LRS and HRS measured up to 300 DC cycles for the PCMO films grown by varying the OP: (**a-1**) 100 mTorr, (**b-1**) 200 mTorr, and (**c-1**) 300 mTorr. Retention characteristics of the LRS and HRS measured up to 10^3 s for the PCMO memristors grown by varying the OP: (**a-2**) 100 mTorr, (**b-2**) 200 mTorr, and (**c-2**) 300 mTorr. (**d**) Retention characteristics of LRS, H_1, H_2, and H_3, measured up to 10^3 s for the PCMO memristor fabricated at 100 mTorr OP.

5. Nonlinear transmission properties of the PCMO memristors fabricated by varying the OP

The synaptic nonlinear transmission properties of the PCMO memristor fabricated by varying the OP were studied. Figure S5a shows the *I-V* curves of the PCMO film grown at 200 mTorr OP with the application of five successive negative voltage sweeps from 0 V to –0.6 V. The current in the PCMO memristor increased negatively with the application of each voltage sweep. However, when five successive positive voltage sweeps varying from 0 V to 1.2 V were applied to the PCMO memristor, the current decreased with the application of each voltage sweep, as shown in Figure S5b. These results clearly show that the current of this PCMO memristor changed with the repeated application of DC bias to the device. Similar results were obtained for the PCMO films grown at 300 mTorr OP, as shown in Figure S5c and d. Because the conduction (or current) of the PCMO memristor is considered as the synaptic weight, continuous changes in the conductance with the repeated application of DC bias can be regarded as the nonlinear transmission characteristic in a biological synapse. Therefore, it can be concluded that the PCMO memristors grown by varying the OP can emulate the nonlinear transmission property of a biological synapse.

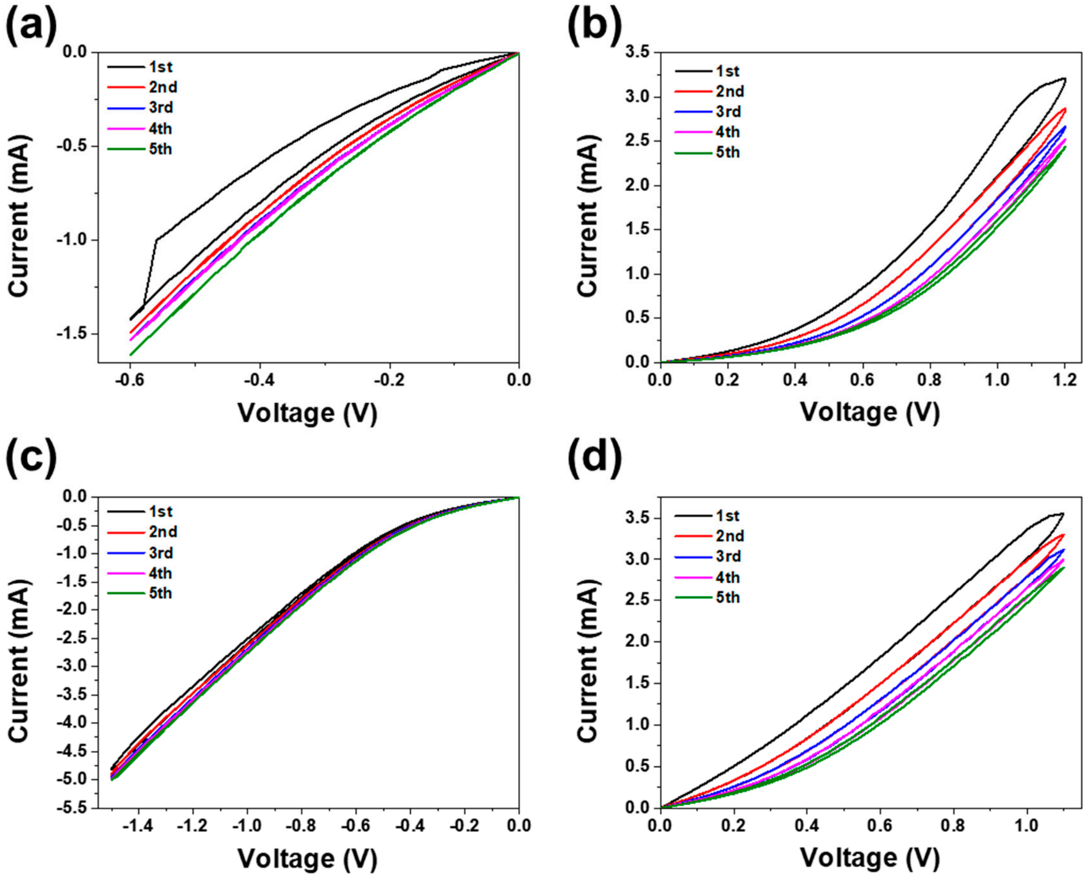

Figure S5. *I-V* curves of the PCMO film grown at 200 mTorr OP measured with the application of five successive (**a**) negative voltage sweeps varying from 0 to –0.6 V and (**b**) positive voltage sweeps varying from 0 to 1.2 V. *I-V* curves of the PCMO film grown at 300 mTorr OP measured with the application of five successive (**c**) negative voltage sweeps varying from 0 to –1.5 V and (**d**) positive voltage sweeps varying from 0 to 1.1 V.

6. Mathematical model to calculate the curvature of the conduction curves and multi-state retention property of PCMO device

Variations in the conductance (G) of RRAM memristors with respect to the pulse number (or time, t) for the potentiation and depression are described by the following equations (S1) and (S2), respectively [3].

$$\frac{dG}{dt}_p = \beta_p \times e^{\left(-c_p \times \frac{G-G_{min}}{G_{max}-G_{min}}\right)} \quad \text{(S1)}$$

$$\frac{dG}{dt}_d = -\beta_d \times e^{\left(-c_d \times \frac{G_{max}-G}{G_{max}-G_{min}}\right)} \quad \text{(S2)}$$

where G_{max} and G_{min} are the maximum and minimum conductance, respectively, of the potentiation (or depression) curve; c_p and c_d are the curvatures of the potentiation and depression curves, respectively; and β_p and β_d are the differences in the conductance between two points on the potentiation and depression curves, respectively. These parameters are illustrated in the potentiation and depression curves (Figure S6a). In addition, the linearity of the conductance modulation of the potentiation (depression) curve is related to the c_p (c_d) value.

The solutions of equations (S1) and (S2) are given by equations (S3) and (S4), respectively:

$$G_{norm,p} = \frac{1}{c_p} \times \ln(w \times e^{c_p} + (1 - w)) \quad \text{(S3)}$$

$$G_{norm,d} = 1 - \frac{1}{c_d} \times \ln(w \times e^{c_d} + (1 - w)) \quad \text{(S4)}$$

where G_{norm} and w are $\frac{G - G_{min}}{G_{max} - G_{min}}$ and $\frac{t}{t_{max}}$, respectively. By using equation (S2), we can express the conductance values of the potentiation curve with respect to the number of pulses for various c_p values, as shown in Figure S6b. When the c_p value is large (10), the curvature of the potentiation curve is considerably large. Hence, this curve exhibits large nonlinear conductance modulation. When the c_p value approaches zero, the linearity of the conductance modulation increases owing to the decrease in the curvature of the potentiation curve (Figure S6b). When the c_p value is zero, the corresponding potentiation curve forms a straight line (Figure S6b). Therefore, the potentiation curve with zero c_p exhibits perfect conductance modulation linearity. In addition, equation (S1) should be used to draw the potentiation curve when c_p is zero, because c_p is in the denominator of equation (S3). Similar results can be obtained for the depression curves, as displayed in Figure S6b. The conduction modulation linearity is therefore concluded to increase when the values of c_p and c_d approach zero. The potentiation and depression curves of the PCMO memristors that were grown by varying the OP can be fitted by using equations (S3) and (S4). Moreover, the c_p and c_d values of the potentiation and depression curves of these memristors can be determined, as shown in Figure 5(a-2)–(c-2).

In addition, the multistate retention of the transmission curve of the PCMO film grown at 100 mTorr was obtained, as shown in Figure S6c. Various number of pulses were applied to this PCMO device, and all states exhibited stable conductance for more than 10^3 s. Therefore, it can be suggested that the PCMO film grown at 100 mTorr OP has good multistate retention of the transmission curve.

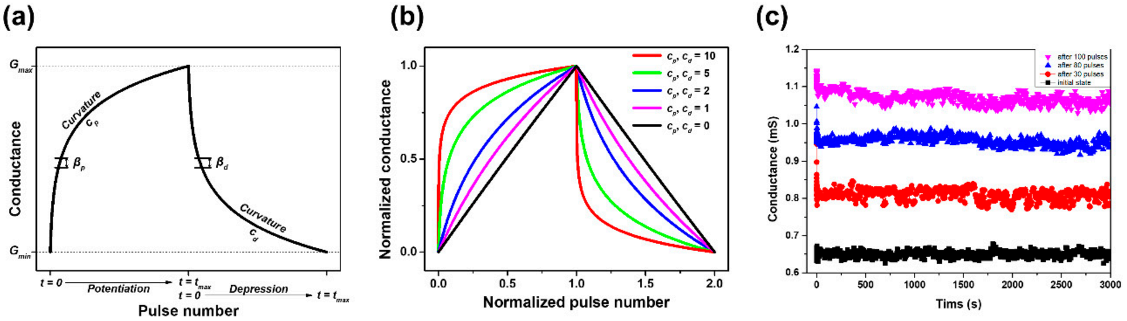

Figure S6. (**a**) Various parameters of equations (S1) and (S2) such as G_{max}, G_{min}, c_p, c_d, β_p, and β_d are indicated on the potentiation and depression curves. (**b**) Potentiation and depression curves with various c_p and c_d values. (**c**) Multistate retention of the PCMO film grown at 100 mTorr obtained from the transmission curve.

7. Detailed convolutional neural network process for pattern classification

A convolutional neural network (CNN) system was used to simulate the digit recognition. The Neural Network Toolbox of MATLAB was employed to operate the CNN model with the PCMO memristor device. The CNN has two components: feature extraction and classification, as shown in Figure S7a.

The feature extraction component, which selects and combines the features of input images, consists of two layers: the convolution and pooling layers. The function of these layers is to simplify the input data and accelerate the learning process. The input pattern consists of digits ranging from 0 to 9 digits from the Modified National Institute of Standards and Technology (MNIST) handwritten digit dataset. Furthermore, this input pattern

has 10,000 images because 1000 images of each digit exist. Each input image consists of 28×28×1 pixels, and these numbers correspond to the height, width, and channel size, respectively. Because the input image is in grayscale, the channel size is 1. In the convolution layer, input images are subjected to the convolution operation via the multiplication of 28×28 image matrices and 3×3 kernel matrices with a stride of 1. The matrices produced from the convolution operation are known as feature maps. A padding process is used to ensure that the size of the output feature maps is the same as the size of the image matrices. Subsequently, batch normalization was conducted to normalize the mean value to 0 and the variance to 1 for each feature map. Batch normalization stabilizes the training process and prevents a change in the distribution. After batch normalization, the rectified linear unit (ReLU) activation function, which is a nonlinear activation function, was used to activate only positive values in the extracted feature map. Successively, the pooling layer follows the ReLU activation function. The feature maps were then subjected to max pooling, which extracts the maximum value from the feature map, and the number of extracted features was reduced. This operation is essential to avoid overfitting problems in CNN simulation. The convolution layer and pooling layer were repeated twice to reduce the amount of computation.

The classification component includes a fully connected layer and the output layer. The softmax function was used for classification in the output layer. In the fully connected layer, the feature map matrix was converted to a vector, which was classified by the neural network. The neural network can be implemented in the form of a crossbar array with the memristor to realize the computation of matrix-vector multiplication. The input vector (x_i) was multiplied by the synaptic weight matrix ($w_{i,j}$). The value of $w_{i,j}$ was defined as $w_{i,j} \equiv g_{i,j}^{+} - g_{i,j}^{-}$, where $g_{i,j}^{+}$ and $g_{i,j}^{-}$ are the normalized conductance of memristors that are close to each other [4]. The values of $g_{i,j}^{+}$ and $g_{i,j}^{-}$ were initialized randomly between g_{min} and g_{max}, where g_{min} and g_{max} are the minimum and maximum values of the normalized conductance, respectively. The output neuron (y_j) is described as $y_j = \sum w_{i,j} x_i$, as displayed in Figure S7b) [5]. The weight between the input and output neurons was updated based on the delta rule: $\Delta w_{i,j} = \eta e_j x_i$ and η is the learning rate and $e_j = t - y_j$ is the error, where t is a target value that is determined using $w_{i,j} = w_{i,j} + \Delta w_{i,j}$ [6]. A neural network was used to calculate the error of the output layer and the weights were updated to minimize the error. The softmax function classifies the output as the class between 0 and 9 using the weighted sum from the crossbar array and presents a probability distribution. The class with the highest probability is determined to be the classification result. The classification process was repeated 20 epochs until saturated accuracy was obtained.

Furthermore, 5-fold cross-validation was used to evaluate the performance of the CNN model using the PCMO memristor. The MNIST dataset consisted of five folds: four training sets and one test set. In the first iteration, the first fold was used as the test set and the remaining 4 folds were used as the training sets. In the second iteration, the second fold was used as the test set while the other four folds were used as training sets. This process was repeated until each of the five folds was used as the test set, as displayed in Table S1. Three PCMO memristors synthesized under different OPs were used to calculate the accuracy. The PCMO synthesized at 100 mTorr OP had the highest accuracy of 95%. The accuracy decreased as the OP increased, as shown in in Table S1. All the memristors exhibited similar accuracies after each iteration, indicating that the CNN model using the PCMO memristor provides highly reliable results.

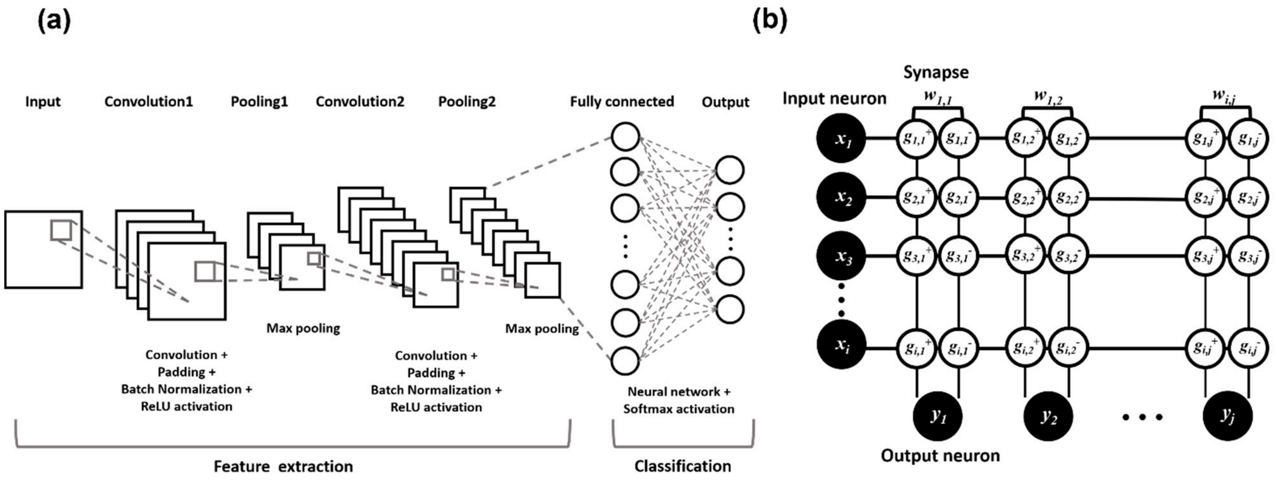

Figure S7. (**a**) Schematic diagram of the basic convolutional neural network (CNN) architecture. (**b**) Diagram of neural networks in the form of crossbar array architecture with memristors.

Table S1. Five-fold cross validation. Comparison of classification accuracies computed by CNNs with weights composed of conductance obtained at 100, 200, and 300 mTorr.

Iteration	Classification accuracy (%)		
	100 mTorr	*200 mTorr*	*300 mTorr*
1	96.25	80.54	70.25
2	91.63	80.41	68.23
3	94.95	77.89	70.50
4	97.81	81.44	69.36
5	96.98	80.81	68.05
**Mean**	**95.52**	**80.22**	**69.28**

8. Modeling of the STP-to-LTP transformation of the PCMO memristor

The forgetful characteristic of the biological brain is generally expressed using the following equation [7,8]:

$$P(t) = P_e + B\exp(-\frac{t}{\tau}) \quad \text{(S5)}$$

where $P(t)$ is a memory level at instant time t and P_e is a memory level obtained after a long time, B is a scaling factor, and τ is a relaxation time constant. A large τ value indicates that a large amount of STP is transformed into LTP. The relaxation curves of the STP-to-LTP transformation for the PCMO memristor were fitted using equation (S5) and P_e and τ were obtained as illustrated in Figure S8a. The P_e increased as the number of P-spikes increased, indicating that the LTP increased with an increase in the number of P-spikes. Therefore, the PCMO memristor can emulate the STM and LTM behavior. Moreover, the τ value also increased when the number of P-spikes increased, as illustrated in Figure S8b, confirming that the amount of STP that was transformed into LTP increased with the number of P-spikes. Therefore, it can be concluded that the STP-to-LTP transformation corresponds well with the memory-loss property of the human brain.

The STP-to-LTP transformation can be explained by the variation in the OV filaments. When the PCMO memristor was transformed from STP to LTP, the current changed from 105 to 135 μA at the reading voltage of 0.1 V, indicating that the STP-to-LTP transformation in the PCMO memristor occurred in the HRS. Hence, the STP-to-LTP transformation of the PCMO memristor can be explained by the change in the length of the OV filaments. Figure S8a shows a schematic diagram of the filament formed

in the PCMO memristor in the HRS. When a small number of P-spikes (five P-spikes) were applied to the PCMO memristor, a few OVs moved into this pre-existing filament and increased its length, as shown in Figure S8(b-1); this resulted in an instantaneous increase in current corresponding to the STP. However, these OVs immediately diffused back after the P-spikes stopped because of the concentration gradient of OVs in the PCMO film and few OVs remained in the filament, as shown in Figure S8(b-2). The current then significantly decreased after removal of the P-spikes. Therefore, the current in LTP could be small. The behavior of the OVs, shown in Figure S8(b-1) and (b-2) can explain the STP-to-LTP transformation with the application of a small number of P-spikes.

In contrast, the continuous application of 80 spikes to the PCMO memristor caused many OVs to move into the filament (Figure S8(c-1)), where they remained after removal of the P-spikes. This is because sufficient electrical energy was continuously supplied to the OVs during the application of the 80 P-spikes. Finally, these additional OVs resulted in the growth of the filament, leading to an increase in the filament length, even after the P-spikes stopped (Figure S8(c-2)). Therefore, the increase in the current was permanent, and this condition corresponded to a new stable state, suggesting that the increased LTP current with the application of many P-spikes can be explained by the increase in the length of the OV filaments.

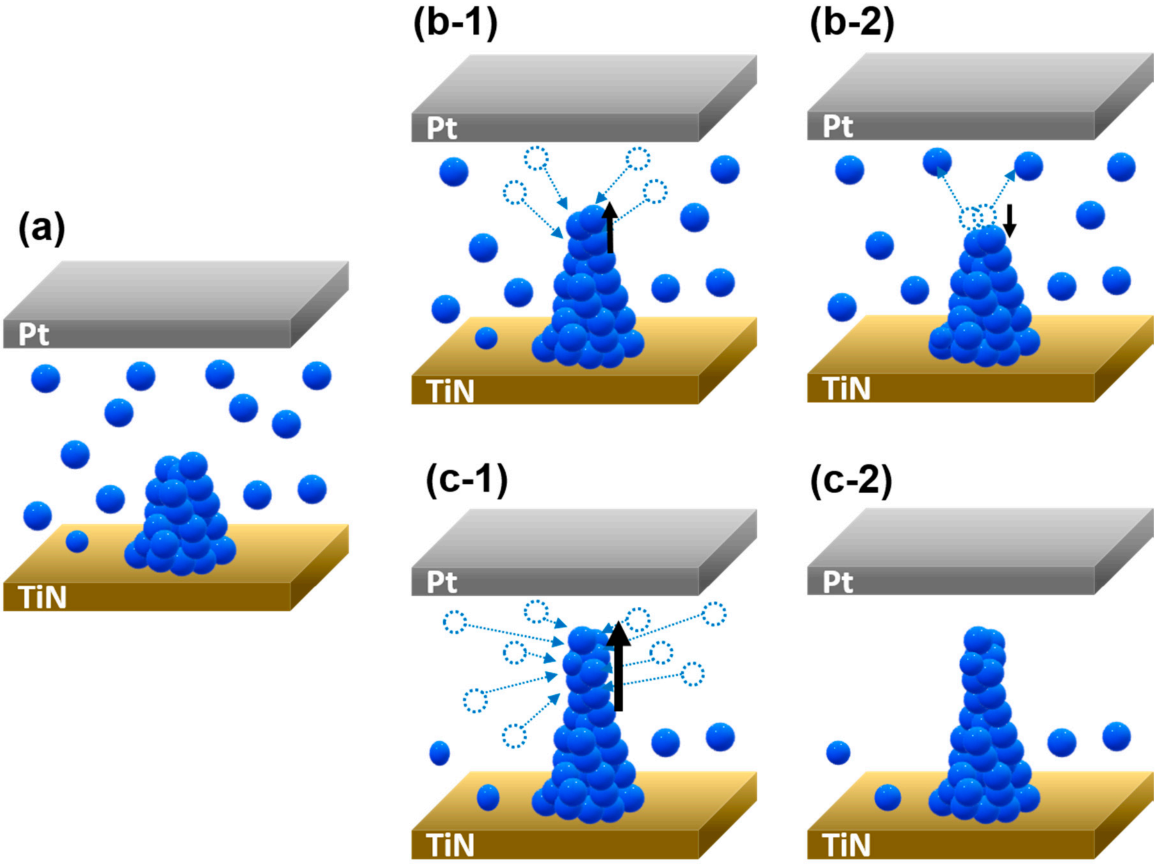

Figure S8. Schematics of the filament length in the PCMO memristor in (**a**) HRS, (**b-1**) with the application of five P-spikes, (**b-2**) after removal of the P-spikes, (**c-1**) with the application of 80 P-spikes, and (**c-2**) after removal of the P-spikes.

9. Various net-spikes used to obtain the synaptic weight change of a PCMO memristor

The pre-synaptic spike consisted of six spikes: five spikes with a positive voltage whose amplitude increased gradually from 0.65 to 0.85 V and one spike with a negative voltage of −0.3 V, as shown in Figure S9a. The post-synaptic spike is the same as the pre-

synaptic spike except that it is a negative spike with a voltage of −0.8 V (Figure S9b). The net spikes applied to the PCMO memristors are the difference between the pre-spike and post-spike, $[V_{pre}(t) - V_{post}(t)]$. Figure S9c shows the various net-spikes that were obtained at different Δt and these net-spikes were applied to the PCMO memristors to determine the synaptic weight change (Δw) with respect to Δt (Figure 8d).

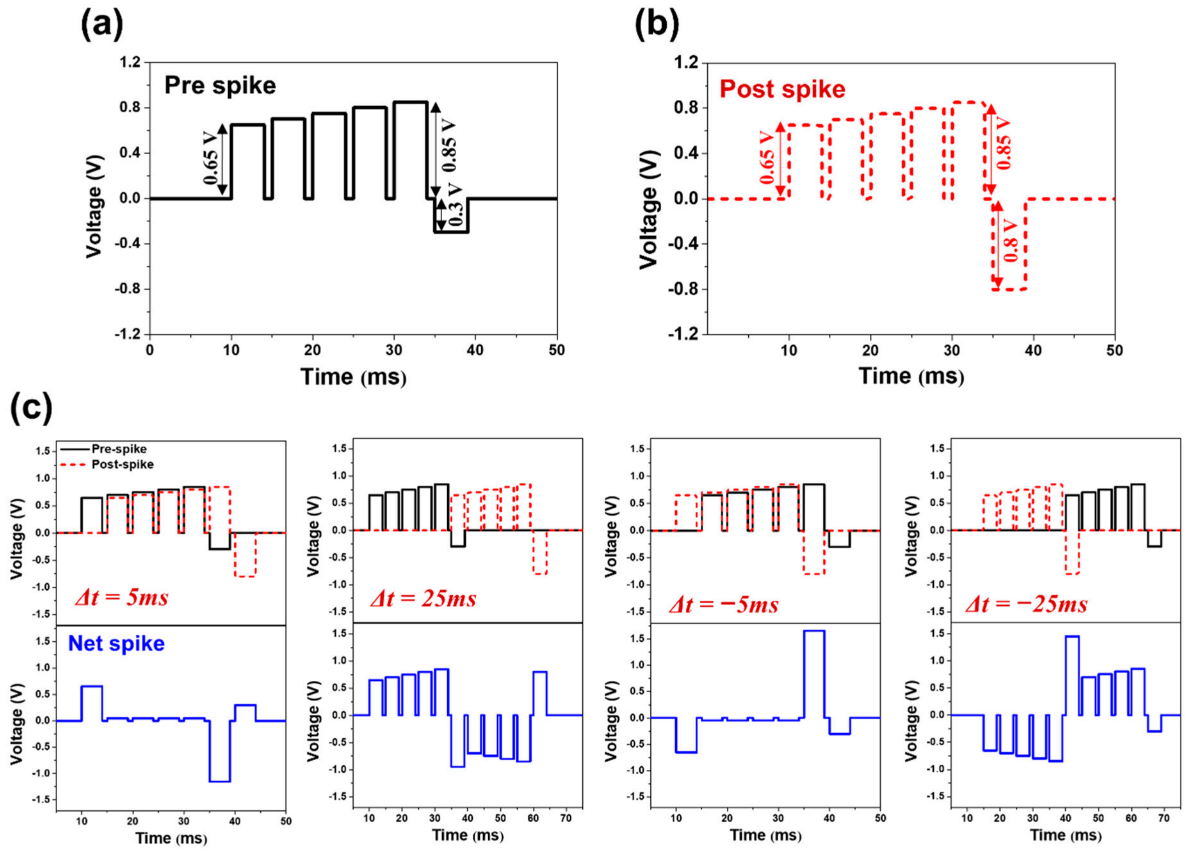

Figure S9. (**a**) Pre-, (**b**) post-, and (**c**) various net-spikes applied to the PCMO memristors to obtain the synaptic weight change (Δw).

References

[1] W.-Y. Chang, J.-H. Liao, Y.-S. Lo, T.-B. Wu, *Appl. Phys. Lett.* **2009**, *94*, 172107
[2] Campbell DS, Maissel L, Glang R, Handbook of thin film technology, McGraw-Hill, New York, 1970; 12: 3
[3] D. Querlioz, P. Dollfus, O. Bichler, C. Gamra, Learning with memristive devices: How should we model their behavior?, 2011 IEEE/ACM Int. Symp. Nanoscale Archit., 2011; 150–156.
[4] E. Zamanidoost, F. M. Bayat, D. Strukov, I. Kataeva, Manhattan rule training for memristive crossbar circuit pattern classifiers, Proc. 2015 IEEE 9th Int. Symp. Intell. Signal Process. (WISP), 2015; 1–6.
[5] F. Alibart, L. Gao, B. D. Hoskins, D. B. Strukov, *Nanotechnology* **2012**, *23*, 075201.
[6] J. L. McClelland, D. E. Rumelhart, P. R. Group, *Explorations in the Microstructure of Cognition* **1986**, *2*, 216–271
[7] A. Watt, N. Desai, *Front. Synaptic Neurosci.* **2010**, *2*, 5.
[8] S. Song, K. D. Miller, L. F. Abbott, *Nature Neurosci.* **2000**, *3*, 919–926
